# Bacteriophages in the treatment of cutaneous infections and skin disorders: therapeutic advances and future directions

**DOI:** 10.1128/cmr.00048-26

**Published:** 2026-06-10

**Authors:** Emilia Hauza, Ivy J. Mutai, Agnieszka Necel, Deus Kamya, Paulina Śliwka, Izabela Dusza, Alicja Węgrzyn, Aneta Skaradzińska

**Affiliations:** 1 Department of Biotechnology and Food Microbiology, Faculty of Biotechnology and Food Science, Wrocław University of Environmental and Life Sciences 56641, Wrocław, Poland; 2 Division of Microbiology and Infection, Becky Mayer Centre for Phage Research, University of Leicester, Leicester, United Kingdom; 3 Antimicrobial Resistance & Bacteriophage Biology Section, Kenya Institute of Primate Research 357902, Karen, Nairobi, Kenya; 4 Department of Medical Microbiology, Faculty of Medicine, Medical University of Gdańsk 37804, Gdańsk, Poland; 5 University of East Anglia, Research Park 6106 , Norwich, United Kingdom; 6 Quadram Institute, Research Park 7308, Norwich, United Kingdom; 7 Centre for Microbial Interactions, Research Park 56641, Norwich, United Kingdom; 8 Phage Therapy Center, University Center for Applied and Interdisciplinary Research, University of Gdańsk 49646, Gdańsk, Poland; Nova Southeastern University, Fort Lauderdale, Florida, USA

**Keywords:** bacteriophages, bacteriophage therapy, skin and soft tissue infections (SSTIs), chronic wounds, antibiotic resistance, melanoma

## Abstract

Skin and soft tissue infections represent a major clinical challenge. This is particularly true given the rise in antimicrobial resistance, the increasing prevalence of chronic wounds, and the growing number of immunocompromised patients. Conventional antibiotic therapies are frequently compromised by multidrug-resistant pathogens, biofilm formation, and disruption of the skin microbiome, underscoring the urgent need for alternative or adjunctive antibacterial strategies. Bacteriophages, viruses that specifically infect and lyse bacteria, have re-emerged as promising therapeutic agents due to their high specificity, activity against antibiotic-resistant strains, and capacity to target biofilm-associated infections. This review provides a comprehensive overview of current advances in bacteriophage-based approaches for the treatment of cutaneous infections and skin disorders. We discuss the biological principles of phage therapy, its advantages and limitations, and the regulatory and manufacturing challenges associated with clinical translation. Particular emphasis is placed on topical and biomaterial-based phage delivery platforms, including hydrogels, nanocarriers, and adhesive wound dressings, designed to enhance phage stability, local bioavailability, and therapeutic efficacy. Furthermore, we summarize experimental and clinical evidence supporting the use of bacteriophages against the most clinically relevant skin pathogens. By integrating data from *in vitro* studies, animal models, clinical trials, and compassionate-use cases, this review highlights both the therapeutic potential and current limitations of phage-based interventions in dermatology. Collectively, the available evidence supports the use of bacteriophages as a viable component of future precision antimicrobial therapies for skin infections, while emphasizing the need for well-designed clinical trials, standardized production protocols, and optimized delivery systems to enable broader clinical adoption.

## INTRODUCTION

The skin serves as a natural protective barrier for the body, but it can become susceptible to infections due to injuries, immune system disorders, excessive moisture, or contamination. The clinical manifestations of skin infections vary widely, ranging from mild lesions to life-threatening conditions (1). Skin and soft tissue infections (SSTIs) pose a particular clinical challenge in immunocompromised patients. These individuals are highly susceptible to infections caused by both common and antibiotic-resistant pathogens, increasing the risk of complications and mortality (2). The most frequently implicated bacterial pathogens include *Streptococcus* spp.*, Staphylococcus aureus* (including methicillin-resistant *S. aureus*, or MRSA), and gram-negative bacilli, such as *Pseudomonas aeruginosa*.

Because of the high prevalence of antibiotic resistance in such pathogens, bacteriophages (phages)—viruses that selectively infect and kill bacteria—are increasingly being investigated as potential alternatives. Phage therapy originated in France in the early 20th century and initially attracted significant interest (3). However, the rapid development of antibiotics, along with the global political situation, led to the marginalization of this therapeutic approach, which was practiced primarily in Poland and the countries of the former Soviet Union. Currently, renewed interest in the application of phages is observed, considering both the prevention and treatment of infections caused by bacteria (4).

Skin infections represent one of the predominant groups of infections, with *S. aureus* and *P. aeruginosa* identified as the most frequently isolated opportunistic pathogens from patients (5). Therefore, this review focuses on the potential use of phages against the most relevant skin pathogens. In the following sections, we provide an overview of recent studies exploring the application of phages against specific skin pathogens, highlight progress in clinical research, and discuss commercially available phage-based preparations. While acknowledging current limitations, we underscore the substantial promise that bacteriophage-based strategies hold for the treatment of skin infections. Beyond their antibacterial activity, recent advances in phage delivery systems, their immunomodulatory and wound-healing effects, as well as novel dermatological applications, including acne and melanoma-related phage technologies, are also addressed. This expanded scope reflects the growing recognition of bacteriophages as multifunctional tools in skin health and disease.

## BACTERIOPHAGES AND PHAGE THERAPY

Bacteriophages are viruses that replicate within bacterial cells, leading to the selective elimination of their hosts. Due to this unique biological specificity, these obligatory bacterial parasites or parasitoids (i.e., parasites that ultimately kill their host after exploiting its cellular resources) have attracted increasing interest as tools for controlling bacterial populations (6). Phage therapy refers to the use of bacteriophages as an alternative or adjunctive approach to combat bacterial infections, relying on their ability to target selectively and eliminate specific bacterial hosts. One of the main advantages of phage therapy is the narrow and highly specific host range of bacteriophages (7). The activity of individual phages is typically restricted to a limited number of strains within the same bacterial species (8). In contrast to antibiotics, which often affect entire microbial communities, phages selectively target and eliminate only those bacterial strains to which they can adsorb and replicate within. This targeted action reduces the risk of depleting beneficial microorganisms, the overgrowth of opportunistic pathogens, and the emergence of antimicrobial resistance (9). Maintaining microbiome homeostasis is particularly important in the skin environment, where commensal bacteria help protect against pathogenic colonization; however, it should also be acknowledged that, under certain conditions, they may delay wound healing (10).

A distinctive and particularly advantageous feature of phages is their capacity for so-called “auto-dosing,” defined as their ability to replicate as long as susceptible bacteria are present at the site of infection. This intrinsic amplification mechanism implies that lower initial doses of phage preparations may be required compared to conventional antimicrobial agents (11). In addition to their specificity of action and safety, phages offer other notable benefits over antibiotics, including easier isolation and the potential for genetic engineering to enhance desired therapeutic properties (9). The often-suggested advantage of low production costs for phage therapies (12) should be interpreted with caution, as large-scale manufacturing must comply with stringent Good Manufacturing Practice (GMP) standards imposed by major regulatory agencies such as the European Medicines Agency (EMA) and the U.S. Food and Drug Administration (FDA), substantially increasing overall manufacturing costs (13). Although compassionate use programs and the FDA Investigational New Drug pathway facilitate limited clinical application, they do not eliminate the logistical and financial burdens associated with GMP-compliant manufacturing required for broader clinical deployment (14). It should also be noted that bacteriophage-based products have been successfully authorized for non-human use, including preparations approved by the European Food Safety Authority (EFSA) for controlling bacterial pathogens in animal production (15). However, these approvals fall outside the regulatory framework governing human therapeutics. Consequently, challenges similar to those faced by other biologics, including quality control and scalable production, remain major barriers to the widespread clinical implementation of phage therapy (16–18).

On the contrary, some limitations of phage therapy should be noted. Firstly, the narrow host range—although one of the greatest advantages of phages—is also one of its main limitations. The high specificity of phages necessitates personalized therapy, in which an active phage is precisely selected for the infecting strain or a phage cocktail is applied, which allows for broadening the activity spectrum of the preparation. Furthermore, applied phages must be genetically characterized in detail, since there is a risk of the presence of genes encoding integrases, antibiotic resistance factors, toxins, and other bacterial virulence factors in their genomes. Another critical issue is the need to thoroughly purify phage preparations to remove bacterial endotoxins, lipopolysaccharides (LPS), and proteins, as well as to eliminate potential contamination with temperate phages that may be released from the bacterial strains used during phage production. If inadequately purified, such contaminants may trigger a strong immune response and exacerbate inflammation at the infection site. Importantly, additional purification steps contribute to increased production costs (19, 20). Problems related to the formulation and stabilization of pharmaceutical preparations, the emergence of bacterial resistance to phages, and reduced activity due to the immune system response to bacterial viruses are also frequently listed among the limitations of phage therapy (9).

Nevertheless, the potential of phage therapy has been used for more than a century. It was summarized that between 1922 and 2022, phage therapy was applied to more than 6,300 patients across various medical fields, including pulmonology, urology, gastroenterology, and intensive care, and it was used to treat infections caused by *Enterobacter, Pseudomonas, Staphylococcus, Salmonella*, and many other common pathogenic bacteria (21, 22). Data accumulated over this century of clinical experience indicate not only the safety of phage-based antibacterial strategies but also their considerable therapeutic potential (22). Moreover, despite problems with formal regulations, phage therapy was implemented in many countries in Europe, America, Asia, and Africa (22).

Beyond intact bacteriophages, phage-derived proteins, particularly endolysins, have emerged as an important complementary strategy in the fight against antimicrobial-resistant infections (23). Endolysins are bacteriophage-encoded enzymes that degrade the bacterial cell wall from within during the lytic cycle (24). When applied exogenously, they can lyse gram-positive bacteria rapidly and with high specificity (25), and engineered variants, so-called artilysins, have demonstrated activity also against gram-negative pathogens (26). In the context of cutaneous and soft tissue infections, endolysins offer several practical advantages over intact phages: they are not subject to bacterial resistance mechanisms targeting phage adsorption, they do not carry genetic material, and their production and quality control may be more straightforward within existing biopharmaceutical frameworks (27, 28). Several endolysins, active against major skin pathogens, have demonstrated promising antibacterial and antibiofilm activity in preclinical models, positioning them as attractive candidates for topical dermatological applications (29–31). Together, bacteriophages and their derived enzymes represent a growing arsenal of targeted antibacterial tools, whose application in cutaneous and soft tissue infections is explored in detail in the following sections.

## PHAGE DELIVERY PLATFORMS FOR THE TREATMENT OF CUTANEOUS DISEASES AND WOUND INFECTIONS

**Fig 1:**
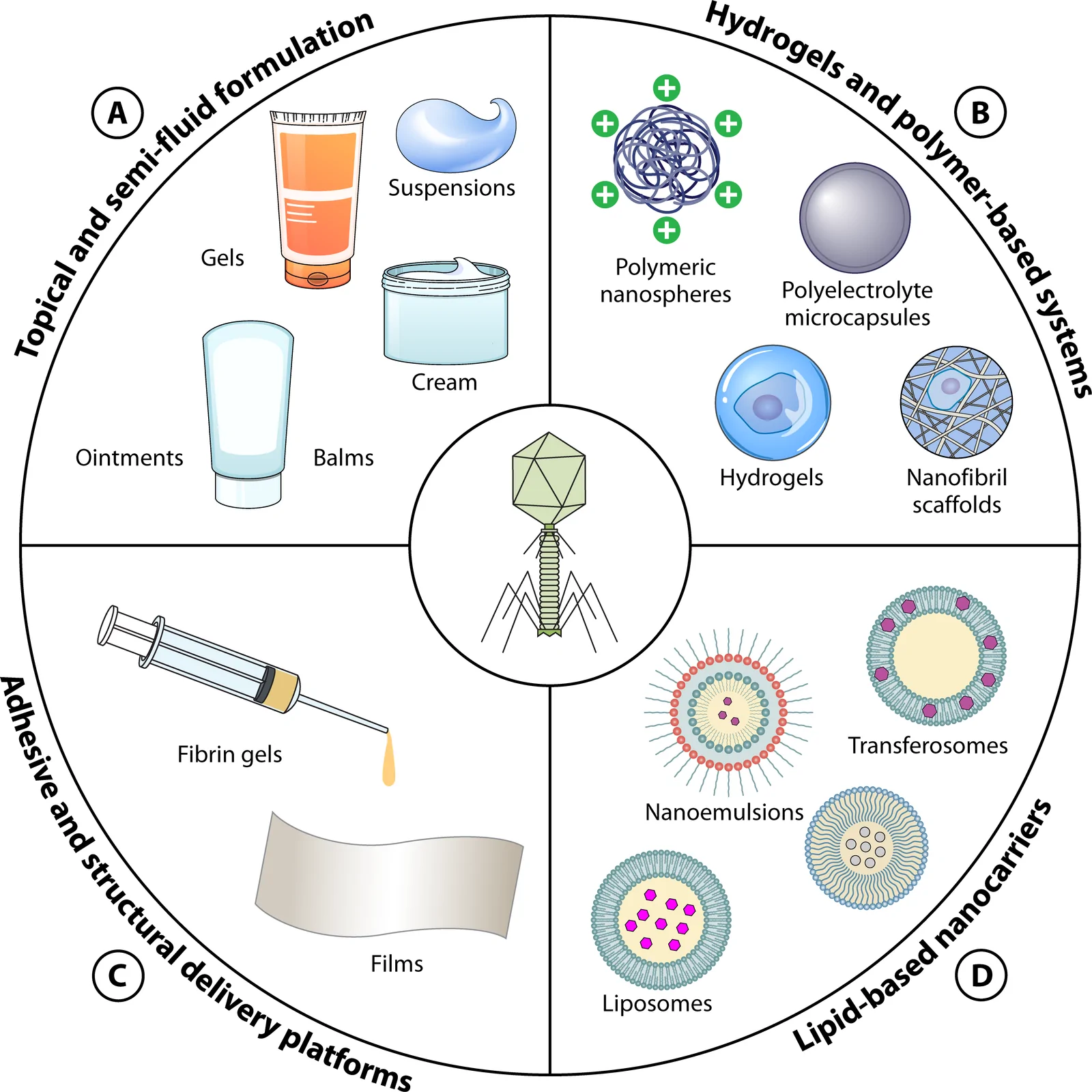
Phage delivery platforms for cutaneous and wound infections. Schematic representation of four main types of bacteriophage delivery systems: (**A**) topical and semi-fluid formulations; (**B**) polymer-based systems; (**C**) adhesive and structural platforms; and (**D**) lipid-based nanocarriers.

Topical administration of phages represents a promising strategy for the treatment of a wide range of skin diseases, including infected wounds, burns, chronic ulcers, post-surgical infections, acne vulgaris, and other bacterial dermatoses (32–38). Due to the complex barrier function of the skin and the presence of bacterial biofilms, effective phage therapy requires delivery systems that ensure phage stability, extended residence time, and high local concentrations at the site of infection (39). Consequently, a variety of topical and biomaterial-based delivery platforms have been developed to optimize phage bioavailability and therapeutic efficacy (39). Phage delivery systems used in cutaneous and wound infections can be broadly classified into topical and semi-fluid formulations, adhesive and structural platforms, polymer-based systems, and lipid-based nanocarriers (32). Figure 1 provides a schematic overview of the major classes of phage-delivery platforms employed in dermatological applications. The figure highlights both conventional topical formulations and advanced biomaterial-based systems designed to enhance phage stability, penetration, and sustained release, thereby facilitating the rational selection of delivery strategies tailored to specific skin conditions and therapeutic goals.

Conventional topical formulations such as ointments, creams, gels, and liquid suspensions have been widely explored due to their simplicity and ease of application (40). However, more advanced delivery platforms have been developed to overcome limitations related to phage degradation, poor penetration, and rapid clearance. These include liposomes, transferosomes, nanoemulsions, adhesive films, fibrin glues, and hydrogel-based systems (41, 42). Hydrogels are of particular interest in dermatological phage therapy, as they provide a moist microenvironment that supports wound healing, enhances patient comfort, and limits pathogen proliferation. For instance, alginate-based hydrogels have demonstrated the ability to preserve phage viability for up to 28 days and effectively control *S. aureus* infections in murine wound models (43). Similarly, thermosensitive hydrogels, such as poloxamer 407, enable *in situ* gelation and sustained phage release, maintaining a high local concentration and improving the clearance of multidrug-resistant *A. baumannii* from infected skin lesions (44).

Nanotechnology-based carriers further expand the therapeutic potential of topical phage therapy. Cationic liposomes protect phages from environmental degradation, enhance adhesion to bacterial biofilms, and increase accumulation at infected skin sites, thereby accelerating wound closure and reducing inflammation (45, 46). Transferosomes, liposomes containing edge activators, demonstrate improved skin penetration and soft tissue protection compared to free phages (47). In addition, stimuli-responsive nanospheres enable temperature- or pH-triggered phage release, enabling controlled and targeted bacterial lysis in inflamed or infected skin tissues (48). Innovative approaches, including 3D-printed hydrogels and carrageenan-immobilized phage dressings, have shown sustained antibacterial activity, reduced biofilm formation, and prevention of bacterial resistance development in both acute and chronic skin infections (49, 50). Collectively, these biomaterial-based delivery platforms significantly enhance phage stability, bioavailability, and therapeutic efficacy, underscoring their potential as key components of modern dermatological phage therapy and advanced wound care.

## POTENTIAL APPLICATION OF BACTERIOPHAGES AGAINST THE MOST COMMON PATHOGENS OF SKIN AND SOFT TISSUE

SSTIs are caused by a diverse group of bacterial pathogens that differ in their epidemiology, virulence mechanisms, and patterns of antimicrobial resistance. The increasing prevalence of multidrug-resistant strains, together with the ability of many skin pathogens to form biofilms and persist in chronic wounds, has significantly limited the effectiveness of conventional antibiotic therapy (2). As a result, bacteriophages have gained attention as a promising alternative or adjunctive approach for the treatment of SSTIs, offering high specificity, activity against antibiotic-resistant bacteria, and potential biofilm-disrupting properties (18). This section provides an overview of the current evidence supporting the use of bacteriophages against the most common bacterial pathogens implicated in skin and soft tissue infections. Below, for each pathogen, we summarize its clinical relevance, major therapeutic challenges, and recent experimental and clinical advances in phage-based interventions.

### Staphylococcus aureus

*S. aureus* is one of the most widespread and clinically relevant bacterial pathogens, responsible for a broad spectrum of infections ranging from mild SSTIs to severe, life-threatening invasive diseases (51, 52). In dermatological contexts, *S. aureus* is a leading cause of furuncles, abscesses, wound infections, and post-surgical complications, which, despite often being non-fatal, collectively impose a substantial burden on healthcare systems due to their high prevalence and recurrence rates (53, 54).

A major therapeutic challenge associated with *S. aureus* infections is the rapid emergence and global dissemination of antibiotic-resistant strains. MRSA remains of particular concern, as it is associated with increased mortality, prolonged hospitalization, and limited treatment options across diverse clinical settings (55). In addition to antimicrobial resistance, the capacity of *S. aureus* to form biofilms on damaged skin, wound surfaces, and medical devices further compromises antibiotic efficacy and contributes to chronic and recurrent infections (56). These features highlight the limitations of conventional antimicrobial strategies and underscore the need for alternative or adjunctive therapeutic approaches.

In this context, bacteriophages have attracted growing interest as targeted antibacterial agents against *S. aureus*. It has been demonstrated that lytic phages isolated from environmental and clinical sources exhibit high activity against both methicillin-sensitive (MSSA) and methicillin-resistant strains. For example, Liu et al. (57) isolated four distinct *S. aureus*-specific phages that individually lysed 80%–95% of tested isolates of MSSA and MRSA clinical strains. Importantly, a cocktail composed of these phages (APTC-C-SA01) expanded host-range coverage to over 98% of isolates, illustrating how phage combinations can partially overcome the narrow host specificity that characterizes individual phages (57).

Beyond monotherapy, combining phages with conventional antibiotics has emerged as a promising strategy to enhance antibacterial efficacy. *In vitro* studies have demonstrated synergistic or additive effects against *S. aureus*, particularly in biofilm settings. For example, the combination of phage Sb-1 with daptomycin resulted in a 2 log CFU/mL reduction in MRSA biofilm-associated bacteria compared to either agent alone (58). Similarly, vancomycin combined with phage K reduced MRSA biofilm populations to below 2 log CFU/mL, outperforming monotherapies (59). These findings suggest that phages may restore or potentiate antibiotic activity, especially in infections where biofilm formation limits drug penetration.

Phage-based approaches have also been investigated in inflammatory skin disorders in which *S. aureus* plays a key pathogenic role, such as atopic dermatitis (AD). Shimamori et al. (60) demonstrated *in vitro* that phage SaGU1 effectively lysed a broad range of *S. aureus* isolates from AD patients without affecting *Staphylococcus epidermidis*, a commensal species important for skin homeostasis (60). In a murine AD model, topical application of SaGU1 reduced bacterial burden, lowered plasma IgE levels, and improved histopathological outcomes. Furthermore, combining SaGU1 with *S. epidermidis* suppressed the emergence of phage-resistant *S. aureus* mutants, suggesting that phage-probiotic strategies may help mitigate resistance development (61).

Despite these encouraging results, the role of phages in *S. aureus*-associated skin diseases is complex and not yet fully understood. Comparative genomic analyses indicated that *S. aureus* strains colonizing healthy skin carried significantly more prophages than isolates from AD patients. In contrast, prophages present in clinical strains were more likely to encode virulence factors (62), highlighting the dual nature of phage-bacteria interactions in the skin environment and underscoring the need for careful selection and characterization of therapeutic phages.

### Pseudomonas aeruginosa

*P. aeruginosa* is an opportunistic gram-negative pathogen and one of the most significant causes of both community- and hospital-acquired infections worldwide. Although it is most commonly associated with pulmonary infections, including ventilator-associated pneumonia and chronic lung infections in cystic fibrosis patients, *P. aeruginosa* also plays an important role in SSTIs, particularly in burn wounds, postoperative sites, and chronic ulcers (63, 64). Global surveillance studies indicated that *P. aeruginosa* is among the most frequently isolated pathogens from chronic wound infections, ranked second after *S. aureus* in many regions (65).

The clinical relevance of *P. aeruginosa* in dermatology is largely driven by its exceptional capacity to persist in hostile environments. This pathogen readily forms robust biofilms on damaged skin and wound surfaces, which protect bacterial cells from host immune responses and significantly reduce susceptibility to antibiotics (66). Moreover, *P. aeruginosa* exhibits a remarkable ability to acquire and express multiple antibiotic resistance mechanisms, including efflux pumps, reduced membrane permeability, and horizontally acquired resistance genes (67). According to recent European data, 17.9% of *P. aeruginosa* isolates are resistant to at least two classes of antibiotics, while 3.5% show resistance to all five tested groups, including fluoroquinolones, aminoglycosides, carbapenems, cephalosporins, and piperacillin/tazobactam (64). As a member of the ESKAPE (*Enterococcus faecium*, *S. aureus*, *Klebsiella pneumoniae*, *Acinetobacter baumannii*, *P. aeruginosa*, and *Enterobacter* spp.) group of pathogens, *P. aeruginosa* exemplifies the urgent need for alternative antibacterial strategies.

Given these challenges, bacteriophages have been extensively investigated as potential tools to control *P. aeruginosa* skin infections. Numerous *in vitro* and *ex vivo* studies have demonstrated that lytic phages can effectively reduce *P. aeruginosa* populations in planktonic cultures as well as in biofilms formed on abiotic surfaces and skin models. For example, Vieira et al. (68) demonstrated that phage PA709 reduced viable *P. aeruginosa* cells by approximately 5 log units *in vitro* and by 4 log units in human skin samples (68). Similar reductions have been observed in *ex vivo* biofilms formed on porcine skin and *in vitro* on polystyrene surfaces, although the magnitude of effect varies depending on phage type, multiplicity of infection, and biofilm maturity (69).

Animal models further support the potential of phage therapy against *P. aeruginosa* skin infections. In rodent wound models, topical application of phages has consistently led to significant reductions in bacterial load, improved wound parameters, and accelerated healing compared to untreated controls (70, 71). Comparable results were obtained with phage phPS127 in rat models, where treatment resulted in a significant reduction in bacterial load and complete closure of most wounds (72). An overview of representative *in vivo* studies evaluating bacteriophage therapy in skin and wound infection models, including infections caused by *P. aeruginosa,* is presented in Table 1, highlighting experimental design, treatment strategies, and key therapeutic outcomes.

**TABLE 1:** Overview of research on phage-based approaches for controlling skin pathogens in *in vivo* models

Model (reference)	Experimental group	Animals (number, species)	Induction of infection/disease	Intervention (type, dose, regimen)	Observation time/measurement points	Assessed parameters (endpoints)	Key results
*P. aeruginosa*, wound (73)	Healthy control (C)	5 BALB/c mice (~25 g)	No infection	No treatment	Days 1, 3, 7, 10, 14	Wound healing, skin condition, histology	Skin almost fully regenerated; no inflammation
	Infected untreated (B)	10 mice	Inoculation with *P. aeruginosa*	No treatment	Days 1, 3, 7, 10, 14	CFU, survival, healing, histology	High bacterial load; ~60% mortality; delayed healing; strong fibrosis
	Hydrogel without phages (H)	5 mice	Inoculation with *P. aeruginosa*	Hydrogel only, daily	Days 1, 3, 7, 10, 14	CFU, survival, healing, histology	Persistent infection; marked inflammation and fibrosis
	Phage hydrogel daily (PD)/once (PO)	5 mice per group	Inoculation with *P. aeruginosa*	Hydrogel + phage cocktail, daily/single application	Days 1, 3, 7, 10, 14	CFU, survival, healing, histology	Rapid bacterial clearance; almost complete skin regeneration; daily > single
	Antibiotic hydrogel (AD/AO)	5 mice per group	Inoculation with *P. aeruginosa*	Hydrogel + ciprofloxacin, daily/single	Days 1, 3, 7, 10, 14	CFU, survival, healing, histology	Good bacterial reduction; slower healing vs phages; single < daily
	Combination phage + antibiotic (PAD/PAO)	5 mice per group	Inoculation with *P. aeruginosa*	Hydrogel with phages + ciprofloxacin, daily/single	Days 1, 3, 7, 10, 14	CFU, survival, healing, histology	Best outcomes; rapid healing; full skin layer restoration
*S. aureus* SAP71, AD model (74)	Healthy control (CT)	Mice	No infection	No treatment	Measurement points after AD + infection/treatment	Skin thickness, clinical changes, histology, IgE, *S. aureus* CFU	Normal skin; low IgE; no significant bacterial burden
	AD + *S. aureus* (AD group)	Mice	DNCB* sensitization + *S. aureus* infection *DNCB - 2,4-dinitrochlorobenzene	No treatment	Measurement points after AD + infection/treatment	Skin thickness, clinical changes, histology, IgE, *S. aureus*	Skin thickening; high IgE; heavy bacterial burden; severe AD symptoms
	Phage treatment (PT)	Mice	As above	Phage SAP71, topical; PFU dose + regimen per article	Measurement points after AD + infection/treatment	Skin thickness, clinical changes, histology, IgE, *S. aureus*	Marked bacterial reduction; decreased skin thickness and IgE; improved histology and clinical symptoms
SaGU1 + *S. epidermidis*, *S. aureus* + AD (61)	AD untreated	Mice BALB/c, *n* = 7 per group	DNCB sensitization + *S. aureus* SA-1 (10^5^ CFU/mL)	No therapy	Days 0, 1, 3, 5 (CFU d1; IgE d3; histology d5)	Skin CFU, IgE, histology (epidermal thickness)	~3,000 CFU; ~3× higher IgE vs control; epidermal thickening and histological changes
	Phage SaGU1	Mice BALB/c, *n* = 7	DNCB sensitization + *S. aureus* SA-1 (10^5^ CFU/mL)	Phage SaGU1 (10^8^ PFU/mL, MOI = 1,000), topical 1 h post infection	Days 0, 1, 3, 5 (CFU d1; IgE d3; histology d5)	Skin CFU, IgE, histology (epidermal thickness)	CFU ↓ from ~3,000 to ~24 (day 1); IgE ↓ ~53% (day 3); histology and thickness improved; reduced epidermal hyperplasia
	*S. epidermidis* treatment	mice BALB/c, *n* = 7	DNCB sensitization + *S. aureus* SA-1 (10^5^ CFU/mL)	*S. epidermidis* SE-4 (10^7^ CFU/mL)	Days 0, 1, 3, 5 (CFU d1; IgE d3; histology d5)	Skin CFU, IgE, histology (epidermal thickness)	Reduced IgE; decreased *S. aureus;* histology improved; milder symptoms vs untreated
	Combination phage + *S. epidermidis*	Mice BALB/c, *n* = 7	DNCB sensitization + *S. aureus* SA-1 (10^5^ CFU/mL)	SaGU1 + SE-4 co-applied	Days 0, 1, 3, 5 (CFU d1; IgE d3; histology d5)	Skin CFU, IgE, histology (epidermal thickness)	Similar *S. aureus* reduction as phage alone; recovery of phage-sensitive strains; no strong synergy but improved immune/histology profile
*A baumannii* K9, sepsis, and burn skin infection (75)	Control + treatment groups	BALB/c mice, female, 9–10 weeks, 18–20 g; *n* = 10 per group	Sepsis: i.p.* challenge with *A. baumannii* B05 (6.3 × 10^5^ CFU/mL, 40 LD_50_) in mucin; Burn model: thermal injury + topical infection with A. baumannii *Intraperitoneal provocation	Phage AM24 (10^9^ PFU/mL mouse, i.p. or topical) OR depolymerase DepAPK09 (50 µg/mouse) preventive (1 h before infection), early (1.5 h post), delayed (6 h post); in burn model: topical application 24 h post-infection, repeated twice at 6 h interval	Sepsis: survival up to 10 days; organ and blood sampling after sacrifice; Burn: bacterial counts 48 h post-infection	Survival; bacterial burden (blood, organs, wound surface, deep skin layers); infection signs; phage/enzyme stability (pH, temperature)	Sepsis: 100% survival with preventive or early treatment (phage or enzyme); untreated died ~2 days; delayed treatment: ~70% survival (phage), ~40% (enzyme); survivors free of bacteria in blood/organs. Burn: ~3-fold reduction (surface) and ~10-fold (deep layers) in bacterial load; phage/enzyme stable pH 5–9, 4°C–56°C; inactivated at extreme conditions
*E. coli,* wound infection (76)	Control group	BALB/c mice (female, 6–8 weeks, 23 ± 2 g, *n* = 6)	Anesthetized by intramuscular injections of 50 mg/kg Zoletil 50. The skin on both sides of the thoracodorsal region of mice was excised into 10 mm diameter wounds. Then, the wounds were infected with 50 μL of *E. coli* (108 CFU/mL)	Treated with saline	Day 1, 2, and 3 post-inoculation, final measurement - day 14	Histopathological analysis, including HE staining, Masson staining, and immunohistochemistry staining (IL-1β, TNF-α, and VEGF)	The recovery rate in the normal saline group showed a relatively stable and slow trend, with more invaded lymphocytes and more pro-inflammatory cytokines (IL-1β and TNF-α)
	Treatment group	BALB/c mice (female, 6–8 weeks, 23 ± 2 g, *n* = 6)	Anesthetized by intramuscular injections of 50 mg/kg Zoletil 50. The skin on both sides of the thoracodorsal region of mice was excised into 10 mm diameter wounds. Then, the wounds were infected with 50 μL of *E. coli* (10^8^ CFU/mL)	Treated topically with 50 μL of *Tequatrovirus* phage YZ2 (10^8^ PFU/mL)	Day 1, 2, and 3 post-inoculation, final measurement - day 14	Histopathological analysis, including HE staining, Masson staining, and immunohistochemistry staining (IL-1β, TNF-α, and VEGF)	Higher recovery rate, significant acceleration of wound healing in the phage-treated group. Relatively intact histological characteristics. Recovery of hair follicles. Decreased levels of IL-1β and TNF-α. More vascular endothelial growth factors (VEGF)
*A. baumannii*, skin infection, and sepsis model (77)	Sepsis model Control vs P92 (5 mg/kg, 10 mg/kg)	BALB/c male mice (18–22 g) bacterial inoculation (17 mice per group), and a control group (4 mice)	Systemic infection with *A. baumannii* (injection) 0.1 mL of 5 × 10^8^ CFU/mL	Peptide P92, intraperitoneal five or 10 mg/kg	1, 7, 13 h Mortality was recorded over 100 h	Survival rate Bacterial burden (blood, lung, liver, spleen)	Significant increase in survival with P92 (dose-dependent) Reduced bacterial loads in blood and organs
	Skin infection model Control vs P92 (5 µM, 10 µM)	BALB/c male mice (18–22 g)	Skin wound infected with *A. baumannii* ATCC 19606 was adjusted to 10^9^ CFU/mL, anesthetized with an intraperitoneal injection of 3.5% chloral hydrate at a dosage of 10 µL/g body weight	Topical/local application of P92, 5 or 10 µM	Three times daily, and observations were made after 2 days. Histology and bacterial burden assessed at defined time points post-infection	Biofilm formation Histological changes (HE, Masson staining) SEM observation of wound surface	P92 reduced biofilm formation Improved skin healing and tissue structure compared to the control

It has become increasingly clear that formulation and delivery are critical determinants of phage efficacy, as their therapeutic potential is strongly influenced by the ability to remain stable and biologically active within the wound environment. In experimental models of *P. aeruginosa*-infected wounds, multiple delivery systems have been developed to enhance phage stability, prolong retention at the wound site, and improve overall treatment efficacy. Among these strategies, hydrogel-based formulations have demonstrated particular promise by effectively supporting both antibacterial activity and wound healing processes (78). In a murine wound model, hydrogels loaded with a phage cocktail, alone or in combination with an antibiotic, led to faster wound closure than antibiotic-containing dressings. Complete wound healing was observed by day 14 in phage-treated groups, and histopathological analysis indicated earlier initiation of skin repair processes when phages were present (73).

Electrospun nanofibrous scaffolds represent another strategy aimed at sustained phage delivery and enhanced interaction with the wound environment. In diabetic mouse models of co-infected wounds, a dual phage-loaded polyvinyl alcohol-Eudragit nanofiber matrix achieved complete bacterial clearance and wound healing within 12 days (79). Similar results were obtained in a murine wound model, where mucoadhesive electrospun nanofibrous scaffolds loaded with phage Pɸ-Mi-Pa achieved full wound closure within 7–10 days, in contrast to untreated wounds, which showed no healing over the same period (80).

Despite promising preclinical results, clinical translation of phage therapy for *P. aeruginosa* skin infections has proven challenging. The most widely cited clinical trial, PhagoBurn, was a randomized, double-blind, phase 1/2 study evaluating a phage cocktail (PP1131) for the treatment of burn wound infections (Table 3). The trial was terminated due to insufficient efficacy, which was later attributed not to the intrinsic failure of phage therapy, but to a substantial reduction in phage titer during manufacturing and storage, resulting in doses far below the therapeutic threshold (81). This study, despite including a serious mistake in basic microbiological procedures, underscored the critical importance of stringent quality control, adequate dosing, and formulation stability in clinical applications of phages. In contrast, smaller clinical studies and compassionate-use reports suggested that phage therapy may still offer benefits in selected cases. Gupta et al. (82) reported clinical improvement in patients with chronic, non-healing wounds infected with *P. aeruginosa* following topical phage application. In these patients, wounds that had persisted for more than 6 weeks despite antibiotic treatment became sterile within 9–13 days, accompanied by reductions in wound depth, necrotic tissue, edema, and inflammatory markers. Although limited by small sample size and lack of controls, these findings indicated that phages may contribute to infection control and wound improvement when conventional therapies fail (82).

### Streptococcus pyogenes

*S. pyogenes,* the predominant species carrying the Lancefield group A antigen, is commonly referred to as Group A *Streptococcus* (GAS) (83). This strictly human pathogen colonizes the throat, genital mucosa, rectum, and skin, where it may exist as both a commensal organism and an opportunistic pathogen. Globally, *S. pyogenes* is one of the leading causes of bacterial infections, with an estimated 1.78 million new severe infections annually and approximately 18.1 million prevalent cases worldwide (84). GAS-associated diseases include both invasive and non-invasive conditions, ranging from pharyngitis and impetigo to necrotizing fasciitis and streptococcal toxic shock syndrome. Importantly, SSTIs account for a substantial proportion of GAS-related hospitalizations; a recent multicenter European study reported that approximately 23% of pediatric GAS cases were associated with SSTIs, underscoring the dermatological relevance of this pathogen (85). Unlike several other major skin pathogens, *S. pyogenes* remains largely susceptible to β-lactam antibiotics. Nevertheless, therapeutic challenges persist due to its ability to cause rapidly progressing infections, severe systemic complications, and immune-mediated sequelae such as rheumatic heart disease (86, 87). In low-resource settings, where access to timely antibiotic treatment may be limited, GAS-related skin infections such as impetigo remain highly prevalent, particularly among children (88). These epidemiological and clinical factors motivate continued exploration of alternative or adjunctive antimicrobial strategies, including bacteriophage-based approaches.

The biology of *S. pyogenes*-phage interactions is complex and distinct from that of many other bacterial pathogens. All known *S. pyogenes* strains are lysogenized with one or more prophages, which play a central role in horizontal gene transfer, population structure, and pathogenic potential (89). Most GAS phages are temperate and capable of generalized transduction, complicating their direct therapeutic application. Furthermore, the limited number of fully sequenced *S. pyogenes* phages available in public databases restricts comprehensive assessment of their genomic diversity and safety profiles (90, 91).

Despite these challenges, several lytic or predominantly lytic phages targeting *S. pyogenes* have been identified and characterized. Bacteriophage A25 (also known as phage 12204) remains the most extensively studied example. Although originally derived from a lysogenic ancestor, phage A25 has lost its key lysogeny genes and is currently considered functionally lytic. Its genome exhibits a modular organization characteristic of GAS prophages, retaining regulatory elements such as a coupled operator-two promoter region and a *cro*-like gene coding for a repressor competitor (antirepressor) (92). Noteworthy is that phage A25, together with closely related historically isolated phages A6, A12, and A27, has contributed substantially to the foundational understanding of GAS-phage interactions, particularly in early studies of phage biology and host specificity. However, many of these phages remain insufficiently characterized by modern genomic standards (93). In contrast, phage A1, initially classified as strictly lytic, is now believed to be a temperate virus with its genome integrated into chromosomes of certain *S. pyogenes* strains, where it confers superinfection immunity (94).

More recently, the putative lytic phage Spy has demonstrated activity not only against *S. pyogenes* but also against other streptococcal species, including *S. parasanguinis, S. agalactiae*, and *S. salivarius*. Notably, this phage remains stable under conditions characteristic of the human body and oral cavity, supporting its potential utility against drug-resistant streptococcal infections (95).

In parallel with academic research, several commercial phage-based preparations targeting *S. pyogenes* have become available, primarily in Eastern Europe and the Caucasus region. These include Pyofag, a multi-component phage cocktail containing phages active against *S. pyogenes* and other bacterial pathogens, as well as Streptophage, a liquid formulation intended for oral, local, or external use in streptococcal infections (96, 97). In addition, broader-spectrum preparations such as Pyobacteriophage and Phygo/Phygospray also exhibit activity against *Streptococcus* species (97–99). Furthermore, Phage Pastilles, a sustained-release phage cocktail developed at this institute, incorporates the novel lytic phage vB_SPY_7, which demonstrates high activity against *S. pyogenes* and stability in prolonged-release formulations (100). The above-mentioned bacteriophage preparations, together with other commercially available phage-based products relevant to dermatology and wound care, are summarized in Table 2. This table highlights differences between medicinal products used under compassionate-use or local regulatory frameworks (e.g., in Georgia or Poland) and dermo-cosmetic products marketed for acne or sensitive skin. Importantly, this comparison illustrates that while some phage preparations have decades of clinical experience and documented case series, most still lack large randomized controlled trials and formal EMA/FDA approval, which remains a key barrier to their broader clinical adoption.

**TABLE 2:** Commercial phage preparations used for wound and skin infection treatment

Reference(s)	Product	Manufacturer/ country	Formulation	Target pathogens	Indications (skin/wounds)	Evidence/notes	Regulatory status	Year of market introduction
(38, 101)	PhagoBioDerm	Intralytix (USA)/Georgia (licensed)	Biodegradable polymer dressing/sheet containing phages	*P. aeruginosa*, *E. coli*, *S. aureus* (as part of pyophage cocktails)	Chronic wounds, ulceration, radiation burns, infected surgical wounds	Clinical use reported in Georgia; case series and reviews describe significant bacterial load reduction and wound healing. Limited RCT data available	Licensed locally in Georgia; not EMA/FDA approved as a drug, used under compassionate protocols	Introduced ca. 2000
(98)	Pyobacteriophage	Eliava Biopreparations (Georgia)	Liquid solution (oral/topical applications available)	*Streptococcus* spp., *Staphylococcus* spp., *E. coli*, *P. aeruginosa, Proteus* spp., *Enterococcus* spp.	Purulent skin and soft tissue infections, infected wounds, burns, prophylaxis in surgery (historical)	Widely used in Georgian phage therapy practice; case reports on diabetic foot ulcers and chronic wound infections show positive outcomes	Commercially available in Georgia; not registered with EMA/FDA	First production in the 1930s
(99, 102)	Septaphage	Biochempharm (Georgia)	Oral tablets and solutions (may be applied locally depending on indication)	*Shigella, Salmonella, E. coli, Proteus* spp.*, Staphylococcus, Pseudomonas, Enterococcus*	Mainly gastrointestinal infections, but also purulent infections and wound infections (per manufacturer’s description)	Evidence primarily from local clinical experience and manufacturer data; no large-scale RCTs published	Commercial product in Georgia; not EMA/FDA approved	No data on the production date
(103)	Phagoderm	Micromir/Microgen (Russia)	Topical gel containing 40–70 phages depending on version	*S. pyogenes, Staphylococcus* spp.*, P. aeruginosa, Proteus* spp.*,* others (broad cocktail)	Acne, infected wounds, post-surgical prophylaxis, ulcers, burns, cracked skin	Clinical data mainly from Russian literature; marketed as both cosmetic and therapeutic gel	Commercially available in Russia and nearby markets; no EMA/FDA approval	Introduced ca. 2011
(104)	Customized Phage Preparations (PTU)	Phage Therapy Unit, Hirszfeld Institute (Poland)	Individualized phage cocktails (wet dressings, topical solutions, oral if needed)	Broad spectrum depending on patient isolate: *Acinetobacter, P. aeruginosa,* MRSA, *E. coli, Klebsiella, Enterobacteriaceae*	Chronic infected wounds, diabetic foot ulcers, post-surgical wound infections, burns	Personalized therapy based on phage susceptibility testing; published case series show good outcomes in refractory infections	Experimental/compassionate use, authorized by national ethics committees; not marketed as an over-the-counter product	Established 2005
(105)	Phortify Probiotic Serum	Phyla (Phi Therapeutics Inc.), USA	Serum	*C. acnes* (RT4, RT5, RT8 strains)	Acne vulgaris; skin microbiome balancing, reduces inflammatory and non-inflammatory lesions	–^*a*^	RCT with ~90 participants (8 weeks). Cosmetic/dermocosmetic product, available OTC	Announced in early 2021
(106)	Ellis Day Balancing & Hydrating Phage Serum	Ellis Day Skin Science (SmartPhage Inc.)	Serum	*C. acnes* (Cutiphage phage blend)	Acne, blemishes, redness, skin microbiome balance	–	12 week clinical trial reported; marketed as non-prescription skin care product	April 2020
(107)	BX001	BiomX	Topical gel (phage cocktail)	*C. acnes* (acne-associated strains)	Mild-to-moderate acne vulgaris	–	Phase 1/2 clinical trials as a cosmetic product; not an EMA/FDA approved drug	Phase 1 completed 2020; Phase 2 initiated 2021
(108)	Phage Glow Skin Spray /Bioactive Gel	Aumed a.s., Czech Republic	Spray and bioactive gel	*S. aureus, C. acnes*	Sensitive, eczema-prone skin, acne-prone skin, itch relief	–	Marketed as dermocosmetic product with bacteriophage component; no formal drug approval	Launched on the market in 2023

^*a*^ –, not applicable.

Despite their availability and lytic activity, a major limitation of commercially available bacteriophage preparations targeting *S. pyogenes* is the absence of publicly accessible experimental or clinical efficacy data, with most published studies focusing on genomic characterization, stability, or *in vitro* activity rather than controlled animal or clinical studies.

### Klebsiella pneumoniae

*K. pneumoniae* is a species of encapsulated gram-negative bacteria that represents a major cause of both community-acquired and nosocomial infections (109). Although it is classically associated with pneumonia, urinary tract infections, and bloodstream infections, *K. pneumoniae* is increasingly emerging in diabetic foot ulcers (DFUs), burn wounds, and surgical-site wounds among other SSTIs (110). These infections are often associated with prolonged hospitalization and poor clinical outcomes, especially in immunocompromised patients.

A defining feature of *K. pneumoniae* is its thick polysaccharide capsule, which plays a central role in immune evasion, resistance to phagocytosis, and protection against antimicrobial agents (111). In recent years, the global spread of multidrug-resistant and carbapenem-resistant *K. pneumoniae* strains has dramatically limited available treatment options. According to surveillance data, resistance to last-resort antibiotics such as carbapenems and colistin is increasingly reported, placing *K. pneumoniae* among the most urgent antibiotic-resistance threats worldwide (112, 113). In the majority of SSTIs, this resistance is often compounded by the pathogen’s propensity to form biofilms (114). In the context of skin infections, these resistance traits complicate the treatment of wound-associated infections and increase the risk of chronicity and systemic dissemination.

Given these challenges, bacteriophages have been explored as potential tools to control *K. pneumoniae* infections. *In vitro* studies have consistently demonstrated the capacity of lytic bacteriophages to effectively reduce *K. pneumoniae* populations in both planktonic cultures and established biofilms. To date, more than 500 lytic phages targeting *K. pneumoniae* have been isolated; however, their therapeutic applicability is frequently constrained by a narrow host range that is largely determined by capsule-type compatibility (115). This biological limitation has driven the development of phage cocktails as well as the *in vitro* evolution of broad-host-range phage mutants to improve coverage across genetically diverse clinical isolates.

Preclinical *in vivo* validation of phage therapy against *K. pneumoniae* SSTIs has predominantly relied on murine wound models. These models typically involve hair removal followed by the induction of a burn or abrasion, after which wounds are inoculated with a selected *K. pneumoniae* strain (116). Treatment groups receive either phage lysates or an antibiotic comparator, most commonly gentamicin. Across multiple studies, phage-treated animals consistently exhibited reductions of 1–3 log units in tissue bacterial burden within 5–7 days, accompanied by accelerated wound contraction and closure (117, 118). Survival outcomes in phage-treated groups generally matched or exceeded those observed with antibiotic therapy, and no systemic inflammation or adverse effects were reported.

Most experimental protocols employ topical, single-dose phage administration, although epicutaneous and intraperitoneal delivery routes, as well as repeated dosing regimens, have also been explored (118, 119). To enhance local phage retention and prolong antibacterial activity, various delivery systems have been developed, including microparticle encapsulation and hydrogel-based carriers. Similar to delivery strategies used for phages targeting other bacterial pathogens, alginate capsules and related formulations have been shown to promote sustained phage release, extend the therapeutic window, and accelerate wound healing compared to free phage application (45, 114, 120). The translational relevance of these murine models, however, remains limited: wound healing in mice occurs predominantly through myofibroblast-driven contraction, whereas re-epithelialization plays a more prominent role in human tissue repair (121, 122). Moreover, most burn and wound models are monomicrobial and maintained under sterile conditions until deliberate bacterial inoculation, failing to recapitulate the polymicrobial, multi-tissue, and immunologically complex environment of clinical infections (123).

Amid these translational limitations, a notable clinical case highlights the potential of personalized phage therapy for *K. pneumoniae*-associated SSTIs involving both bone and overlying soft tissue. In 2016, a trauma patient suffering from a chronic, pandrug-resistant *K. pneumoniae* fracture-related infection had undergone more than 700 days of unsuccessful antibiotic treatment prior to receiving phage therapy. A patient-specific phage (M1), selected for high lytic activity, was subjected to 15 rounds of *in vitro* evolution to broaden its host range and enhance biofilm penetration. Administered locally via catheter in combination with meropenem and ceftazidime, phage M1 led to rapid microbiological clearance, radiological evidence of bone healing, resolution of the soft tissue infection, and sustained remission for over 3 years. Although transient neutralizing antibodies were detected between days 8 and 18, they did not compromise therapeutic efficacy, and no adverse effects were reported (124).

In summary, bacteriophage therapy against *K. pneumoniae* holds promise, particularly in the context of multidrug-resistant wound infections where conventional antibiotics fail. However, capsule diversity, narrow phage host range, and limited clinical validation currently constrain widespread application. Overcoming these limitations will require the development of comprehensive phage libraries, optimization of cocktail formulations, and rigorously designed studies evaluating efficacy in clinically relevant SSTI models.

### Escherichia coli

*E. coli* is a highly diverse bacterial species that plays a complex role in human health and disease. While commensal strains are an integral component of the natural gut microbiota, pathogenic variants are broadly classified into intestinal pathogenic *E. coli* (IPEC) and extraintestinal pathogenic *E. coli* (ExPEC) (125). IPEC strains constitute a major public health concern, such as foodborne pathogens capable of causing diarrheal diseases, colitis, and hemolytic-uremic syndrome (76). In contrast, ExPEC strains are frequently associated with infections outside the intestinal tract, including urinary tract infections, surgical site infections, and burn wound infections (126). In dermatological contexts, ExPEC strains are of particular relevance, as they possess numerous virulence factors that promote colonization, tissue damage, and immune evasion (127). Moreover, *E. coli* is among the most frequently isolated gram-negative pathogens in diabetic foot infections, underscoring its role as a common opportunistic pathogen in chronic skin wounds (128). Importantly, invasive *E. coli* infections originating from poorly controlled skin lesions may progress to bacteremia and sepsis, highlighting the clinical significance of effective local infection control (129).

Despite the frequent involvement of *E. coli* in traumatic and chronic wound infections, research on bacteriophage therapy targeting this pathogen in skin and wound infections remains relatively limited compared to other major skin pathogens (130). One important consideration in this regard is that bacterial eradication alone does not necessarily translate into successful wound healing. Skin repair is a multifactorial process that requires not only elimination of the infectious agent but also coordinated immune modulation and activation of regenerative pathways responsible for tissue restoration (131). Accordingly, while wound healing is inherently a multifactorial process, the potential utility of phages in *E. coli*-infected wounds should be evaluated not only in terms of antibacterial efficacy but also with respect to their indirect effects on wound-healing processes, an aspect discussed in a dedicated section of this review.

Several preclinical studies have explored the therapeutic potential of lytic *E. coli* phages in experimental wound models. Wang et al. (76) isolated the *Tequatrovirus* phage YZ2 from hospital wastewater and demonstrated that topical application of this phage accelerated healing of *E. coli*-infected wounds in mice. Phage treatment reduced bacterial burden and was associated with decreased levels of proinflammatory cytokines, including IL-1β and TNF-α, alongside increased expression of vascular endothelial growth factor (VEGF), suggesting a link between bacterial clearance and improved regenerative signaling (76). These findings support the concept that effective control of *E. coli* infection can positively influence the wound microenvironment.

The importance of formulation and sustained phage delivery has also been highlighted in diabetic wound models. Shiue et al. (132) developed a hydrogel dressing containing a phage cocktail that included an *E. coli*-specific phage. In a diabetic mouse model, application of this dressing resulted in a 37%–79% reduction in bacterial counts on the wound surface and promoted full-thickness wound healing. In addition to antibacterial activity, phage treatment facilitated reconstruction of skin layers, enhanced angiogenesis, and reduced inflammatory responses. Notably, the hydrogel released only a small fraction (1.7%–5.7%) of the embedded phages within 24 hours, indicating controlled release and prolonged local activity (132). Similarly, Narayanan et al. (50) embedded the lytic coliphage vB_Eco2571-YU1 into a carrageenan-based hydrogel, which exhibited strong bacteriolytic activity without detectable development of phage resistance. This formulation was characterized by favorable physicochemical properties, including stability, flexibility, and hemocompatibility, making it a promising alternative to conventional wound dressings (50).

More complex biohybrid delivery systems have also been proposed to address the challenges of chronic wound healing. Biohybrids composed of mesenchymal stem cell aggregates and gelatin microspheres containing *E. coli*-targeting phages have been developed to combine antibacterial activity with regenerative support. By using gelatins with different isoelectric points, phage release kinetics could be modulated, with microspheres exhibiting a basic pH showing rapid phage release and enhanced tissue regeneration. *In vitro* studies demonstrated high antibacterial efficacy together with improved cell viability and metabolic activity in 3D cultures, suggesting that such systems may support both infection control and tissue repair (133).

Clinical data on phage therapy for *E. coli*-associated skin infections remain scarce but provide important safety insights. In a phase I clinical trial involving 42 patients with chronic venous leg ulcers, phages targeting *P. aeruginosa, S. aureus*, and *E. coli* were applied for 12 weeks and compared with saline treatment. No adverse events attributable to phage therapy were reported, confirming the safety of this approach. However, the absence of significant differences in wound-healing outcomes between treatment groups highlights the need for adequately powered phase II trials to assess therapeutic efficacy (134).

Bacteriophage therapy targeting *E. coli* in SSTIs shows promise in preclinical models, particularly when integrated into advanced delivery systems that support sustained antibacterial activity and tissue regeneration. Nevertheless, the limited number of studies, heterogeneity of experimental designs, and paucity of clinical efficacy data currently restrict definitive conclusions regarding its therapeutic value. Further research focusing on clinically relevant wound models and well-designed clinical trials will be essential to clarify the role of phage-based strategies in managing *E. coli*-associated dermatological infections.

### Proteus mirabilis

*Proteus mirabilis* is a gram-negative opportunistic pathogen most commonly occurring in catheter-associated urinary tract infections (CAUTIs); however, it is also isolated from SSTIs, particularly in chronic wounds, diabetic foot ulcers, and burn wounds, where polymicrobial colonization is frequent (135). Although the overall incidence of *P. mirabilis*-associated skin infections is lower than that of *S. aureus* or *Streptococcus* spp., this pathogen can represent a clinically relevant problem in specific patient populations and healthcare settings. Beyond *P. mirabilis*, other members of the genus, including *P. vulgaris* and *P. penneri*, are also capable of causing human infections, albeit less frequently (136). *P. mirabilis* is responsible for 1%–10% of UTIs and 10%–44% of long-term CAUTIs (137). About 20% of hospital-acquired bacteremia cases and 2% of community-acquired bloodstream infections are caused by urinary tract infections (138). The pathogenic potential of *P. mirabilis* is linked to a combination of virulence traits that complicate infection control. These include robust biofilm formation, rapid acquisition of antibiotic resistance, endotoxin production, swarming motility, and urease activity, all of which contribute to persistence on biotic and abiotic surfaces and resistance to conventional antimicrobial therapies (138, 139). In polymicrobial wound environments, these features may further enhance bacterial survival and impede effective treatment, underscoring the need for alternative or adjunctive antimicrobial strategies.

Although relatively few studies have directly examined bacteriophage therapy for *P. mirabilis* in wound infections, available evidence supports its potential as an effective antibacterial approach. Several lytic phages and phage cocktails have demonstrated strong *in vitro* activity against *P. mirabilis*, particularly in biofilm models. A cocktail containing phages Isf-Pm1 and Isf-Pm2 reduced biofilm mass by approximately 65%, an effect attributed primarily to bacterial cell lysis. The observed downregulation of biofilm-associated genes likely reflects reduced bacterial viability rather than direct modulation of gene expression (135). Other anti-*Proteus* phages isolated from environmental sources, including wastewater, have also shown potent bacteriolytic and antibiofilm effects in *in vitro* systems. *Proteus* virus 309 reduced host viability by 99.9%, while silicon-coated phages vB_PmiP_5460 and vB_PmiM_5461 achieved a 1 log reduction in bacterial load over 168 hours under catheterization conditions. Other phages, including Mydo, BigMira, MidiMira, PMP1-PMP3, PM36, and vB_PmiS-Isfahan, consistently demonstrated over 90% reductions in viable bacterial cells within established biofilms, highlighting their potential relevance for biofilm-associated infections (140–145).

Beyond phage selection itself, strategies aimed at enhancing phage efficacy have also been explored. One such approach involves the use of plasma-activated water (PAW), which generates reactive oxygen and nitrogen species. These species induce oxidative stress, leading to damage of cellular components such as DNA, proteins, and lipids, as well as increased membrane permeability. As a result, bacterial cell integrity is compromised, which may facilitate phage adsorption and penetration. Consequently, PAW significantly enhances the antibacterial activity of phage vB_PmiS_PM-CJR against *P. mirabilis* in both planktonic and biofilm states (146). This phage additionally exhibits biofilm depolymerase activity mediated by its tail spike protein, thereby reducing biofilm adherence to surfaces. *In vivo* relevance of this approach was supported by increased survival of *Galleria mellonella* larvae infected with *P. mirabilis*. This effect was demonstrated using a minimum biofilm eradication concentration (MBEC) assay, which determines the lowest concentration of an antimicrobial agent required to eradicate mature biofilms formed on a surface (147). In this method, biofilms are grown on peg surfaces immersed in bacterial culture and subsequently exposed to serial dilutions of the tested agent. After treatment, the pegs are rinsed to remove planktonic cells, and the remaining biofilm is detached, typically by sonication, into recovery medium. Biofilm viability is then assessed by measuring regrowth, for example, using optical density or viable cell counts, and the MBEC is defined as the lowest concentration that prevents detectable regrowth (148).

Several commercial phage-based preparations have been developed that include activity against *P. mirabilis*, primarily as components of broad-spectrum cocktails. These include Pyobacteriophage, Intestiphage, Pyofag, Sextaphage, Phygo, and Phygospray, which have been reported in the context of *P. mirabilis* infections, although they are not species-specific (96, 97). In addition, multiple individual phage formulations targeting *P. mirabilis* have been announced by commercial entities; however, no experimental or clinical efficacy data for these products have been published to date (149).

Bacteriophage-based strategies targeting *P. mirabilis* demonstrate strong *in vitro* antibiofilm activity and benefit from emerging approaches designed to enhance phage performance, such as plasma-activated water and biofilm depolymerase-active tail spike proteins. However, the limited number of studies addressing clinically relevant wound models and the scarcity of *in vivo* data currently restrict a robust assessment of their therapeutic potential in related SSTIs.

## PHAGE THERAPY FOR INFECTED DIABETIC FOOT ULCERS

DFUs represent one of the most severe complications in diabetic patients, significantly reducing quality of life and, in extreme cases, leading to limb amputation or sepsis. It is estimated that approximately 15%–25% of individuals with diabetes will develop a foot ulcer during their lifetime, with 50%–70% of these cases progressing to severe infection (150, 151). These infections are often polymicrobial and frequently involve antibiotic-resistant bacteria, predominantly gram-positive pathogens such as MRSA and gram-negative bacteria like *P. aeruginosa* (152–154). Given the antibiotic resistance crisis and the high costs associated with DFUs treatment management, exploring alternative therapeutic strategies is essential.

Ghanaim et al. (155) investigated the effects of a phage cocktail on DFUs wound infected with *S. aureus, P. aeruginosa, Klebsiella variicola, E. coli,* and *P. mirabilis*. Using a Sprague-Dawley rat model, they found that phage treatment resulted in better wound-healing outcomes than ceftriaxone (155). Similarly, Nir-Paz et al. (156) assessed the safety of phage TP-102 (10^9^ PFU/mL) in diabetic patients with DFUs infected by *S. aureus, P. aeruginosa,* and *A. baumannii*. Results indicated that TP-102 was well-tolerated, and most patients did benefit from the adjunctive phage therapy, reinforcing the potential of this method as a viable DFUs treatment (156). In turn, Jokar et al. (157) demonstrated that a phage cocktail (Stp2, Psp1, Stp1, and Psp2) significantly reduced mortality in subjects infected with *S. aureus* and *P. aeruginosa* exhibiting DFUs symptoms. This combination improved wound healing when administered alongside gentamicin, suggesting that adjunctive phage-antibiotic therapy could be an effective approach for DFUs infections (157).

Research on phage activity against causative agents of diabetic foot has primarily focused on biofilm-associated infections, as biofilms significantly contribute to the antibiotic resistance observed in bacteria isolated from DFUs. Kifelew et al. (158) evaluated two phage cocktails, AB-SA01 (targeting *S. aureus*) and AB-PA01 (targeting *P. aeruginosa*), against biofilm-associated DFUs infections as well as planktonic cultures to provide a comprehensive assessment of phage potency. *In vitro*, 88.7% of *S. aureus* and 92.7% of *P. aeruginosa* isolates were susceptible to the respective cocktails, with individual phages infecting 66% –94.3% of isolates. Moreover, the treatment significantly reduced biofilm biomass regardless of antibiotic resistance or the presence of non-susceptible strains, highlighting the broad host range of the cocktails, their strong lytic activity, and their biofilm-disrupting potential as alternative or adjunctive therapies in DFUs. Importantly, the phage cocktails also demonstrated strong lytic activity against planktonic cells, confirming their efficacy against both biofilm-associated and free-living bacterial forms (158).

Following promising *in vitro* and animal model results, several clinical studies and treatments of individual patients have investigated the application of phages in DFUs. An overview of completed, ongoing, and planned clinical trials evaluating phage therapy for DFUs and SSTIs in humans is provided in Table 3. For instance, in an *in vitro* study, Mohamed et al. (159) isolated and characterized two phages, ϕPAE1 and ϕPAE2, active against *P. aeruginosa* strains obtained from DFUs. When used together as a phage mixture, they exhibited a broad host range, collectively infecting 74% and 58% of clinical isolates, and showed high specificity, without affecting non-target bacterial species (159). Studies by Fish et al. (160, 161) and Nadareishvili et al. (162) reported that topical and oral phage therapy led to clinical improvement in patients with DFUs. In the compassionate-use case series, nine patients with antibiotic-refractory, poorly vascularized toe ulcers infected with *S. aureus* were treated topically with the monophage Sb-1 (160). All infections responded to treatment, and complete ulcer healing was achieved on average within 7 weeks (up to 18 weeks in one severe case), without adverse effects or the need for amputation. A follow-up case study described full recovery of chronic osteomyelitis-associated DFUs after 7 weekly injections of Sb-1 phage, accompanied by radiographic evidence of bone re-ossification and no recurrence of infection during three years of follow-up (161). Similarly, Nadareishvili et al. (162) reported full ulcer closure, resolution of osteomyelitis, and absence of recurrence one year post-treatment after combined oral and topical phage therapy. In a prospective study on chronic, non-healing wounds, over half of the participants had diabetes, and 74% of diabetic patients responded positively to phage therapy, compared to 91% in the non-diabetic group (163). All patients had previously failed antibiotic treatment, suggesting that phage therapy contributed to their clinical improvement. In another study, Young et al. (154) described the application of adjunctive phage therapy targeting *S. aureus* infections in 10 DFU patients at high risk of amputation. Nine out of 10 patients benefited from the treatment, with no adverse effects reported. Phage therapy facilitated infection resolution and limb preservation in most cases, with only one patient requiring amputation due to unresolved osteomyelitis. All these findings highlighted the potential of phage therapy to transform DFU treatment (154).

**TABLE 3:** Summary of registered and ongoing clinical trials evaluating bacteriophage therapy for SSTIs in humans

Reference(s)	Trial name/NCT	Phase	Status	Infection type	Target pathogen(s)	Phage product	Route	Key outcomes
(81)	Phagoburn trial NCT02116010	I/II	Completed (terminated early)	Burn wound infection	*P. aeruginosa, E. coli*	Phage cocktail	Topical	Limited efficacy due to low phage titers
(134, 164)	WPP-201 NCT00663091	I	Completed	Chronic venous leg ulcers	*S. aureus*, *P. aeruginosa, E. coli*	WPP-201 cocktail	Topical	Safe; no negative impact on healing
(156, 165)	REVERSE / TP-102 NCT04803708	I/IIa	Completed	Diabetic foot ulcers (infected and non-infected)	*P. aeruginosa, S. aureus,* and/or *A. baumannii*	TP-102 phage cocktail	Topical	Well tolerated and safe
(166)	PhagoPied NCT02664740	I/II	Recruiting/ongoing	Diabetic foot ulcers (monoinfected)	*S. aureus* (MRSA or MSSA)	Anti-staphylococcal bacteriophage cocktail	Topical	Not provided
(167, 168)	NCT04323475	I	Recruiting/ongoing	Second degree burn wounds	*S. aureus, P. aeruginosa, K. pneumoniae*	Phage cocktail-SPK	Topical	Results not yet available

## BACTERIOPHAGES FOR SKIN CARE

Acne is a widespread chronic inflammatory skin condition that primarily affects teenagers and young adults. Globally, around 80% of adolescents experience acne, with approximately 20% developing moderate-to-severe forms. The prevalence of adult acne is also rising, currently affecting an estimated 40% of the population. While acne is not a life-threatening condition, it often leads to significant psychosocial effects, including low self-esteem, anxiety, social isolation, and depression, which can ultimately lead to suicidal thoughts or attempts (169).

*Cutibacterium acnes* (formerly *Propionibacterium acnes*) is the most common commensal found on human skin; however, specific phylotypes of this bacterium have been associated with the development of acne vulgaris. The widespread and prolonged use of antibiotics in acne treatment contributes to the growing problem of antibiotic resistance in *C. acnes*, which is among the main causes of treatment failures (170).

The first report regarding *P. acnes* phage was identified by Brzin in 1964 (171). Since then, many studies, including recent ones, have demonstrated the activity of phages against this bacterium. For instance, Yu et al. (172) showed that phage application significantly reduced inflammatory papules in a mouse model of acne. Furthermore, the detailed histopathological evaluation of induced lesions revealed that phage-treated mice displayed no clustering infiltration of inflammatory cells in most individuals (172). Similarly, Rimon et al. (37) observed decreased lesion severity (diameter, elevation, and eschar) in phage-treated animals, along with reduced inflammation. Biopsy of murine lesions revealed reduced inflammation in the group treated with phages compared to the control group of untreated animals. As acne is an immune-driven process, inflammatory markers were analyzed, and it was shown that phage treatment may reduce neutrophil migration to lesions, decrease the expression of inflammatory cytokines and chemokines in the lesions, and lower IL-1β levels in the bloodstream (37).

The basis for treating acne lesions with phages also involves certain molecular mechanisms occurring in the host cell. As demonstrated in the rat model, phages may decrease the level of IGF-1, which is proven to be related to acne severity. In addition, *C. acnes* infection activated the PI3K/Akt pathway while simultaneously reducing the activity of the BAD-dependent apoptosis pathway. This indicated that the bacterium contributes to the inhibition of natural cell death, thereby promoting the maintenance of inflammation (173). After the use of phage φPaP11 –13, the activation of the PI3K/Akt pathway was weakened, which restored the balance in the processes of keratinocyte apoptosis and reduced the inflammatory state (173).

Because of the social significance of acne disease, the need to treat this condition has led to the appearance of at least two commercial preparations on the market. PhageGlow and Phyla Acne Skincare constitute a significant step forward in the further commercialization and availability of bacteriophage-based products for patients. Overall, these findings highlight the promising role of bacteriophages as targeted therapeutic agents for acne management. Their specificity against *C. acnes*, combined with the potential to modulate host inflammatory responses, positions phage therapy as a compelling alternative or adjunct to conventional antibiotic treatments. The emergence of commercial phage-based skincare products further underscores the translational potential of this approach for clinical and cosmetic applications.

## BACTERIOPHAGES IN WOUND HEALING AND TISSUE REGENERATION

Wound healing is a complex, multistage process comprising inflammatory, proliferative, and remodeling phases. During the initial inflammatory phase, neutrophils, macrophages, and dendritic cells are recruited to the wound site and release cytokines and chemokines that contribute to pathogen clearance and tissue debridement. However, persistent or dysregulated inflammation can damage surrounding tissue and impair wound closure, a key feature of chronic wounds such as those associated with diabetes (174). In this context, phages have emerged as potential adjuncts in wound management due to their combined antimicrobial, immunomodulatory, and regenerative effects (175, 176).

Two complementary mechanisms are primarily involved in mediating the effects of bacteriophages on infection control and tissue repair during wound healing. First, they can selectively reduce pathogenic bacterial populations and disrupt biofilms, thereby lowering the inflammatory burden and potentially restoring immune homeostasis (35). Second, there is growing experimental evidence that phages can modulate immune responses: several studies report reduced production of pro-inflammatory cytokines (e.g., TNF-α, IL-6) and increased secretion of anti-inflammatory mediators such as IL-10, which may promote a shift toward a reparative macrophage phenotype (177–179). Consistent with these findings, Cianci et al. (180) discussed that phages may interact with immune cells, including macrophages and dendritic cells, through pattern-recognition receptors such as Toll-like receptors and intracellular nucleic acid sensors. These interactions have been proposed to attenuate excessive inflammatory signaling and favor anti-inflammatory pathways, thereby potentially creating a wound microenvironment more conducive to tissue repair and mitigating infection-associated delays in wound closure (180). Furthermore, some studies suggest that phages may influence neutrophil migration and limit tissue damage caused by reactive oxygen species and proteolytic enzymes. However, these effects appear to be phage- and context-dependent (177). Importantly, phages typically display high specificity for their bacterial hosts and are therefore less likely than broad-spectrum antibiotics to disrupt commensal microorganisms, supporting a more balanced wound microbiome and limiting dysbiosis (181, 182).

*In vivo* and preclinical studies further support these observations, showing accelerated wound closure and decreased bacterial load in animal models following phage administration (176). However, it is also important to note that not all phages have uniformly beneficial effects: some filamentous phages, such as the Pf phage from *P. aeruginosa*, have been shown to delay wound healing by interfering with immune signaling and reducing bacterial clearance (183).

**Fig 2:**
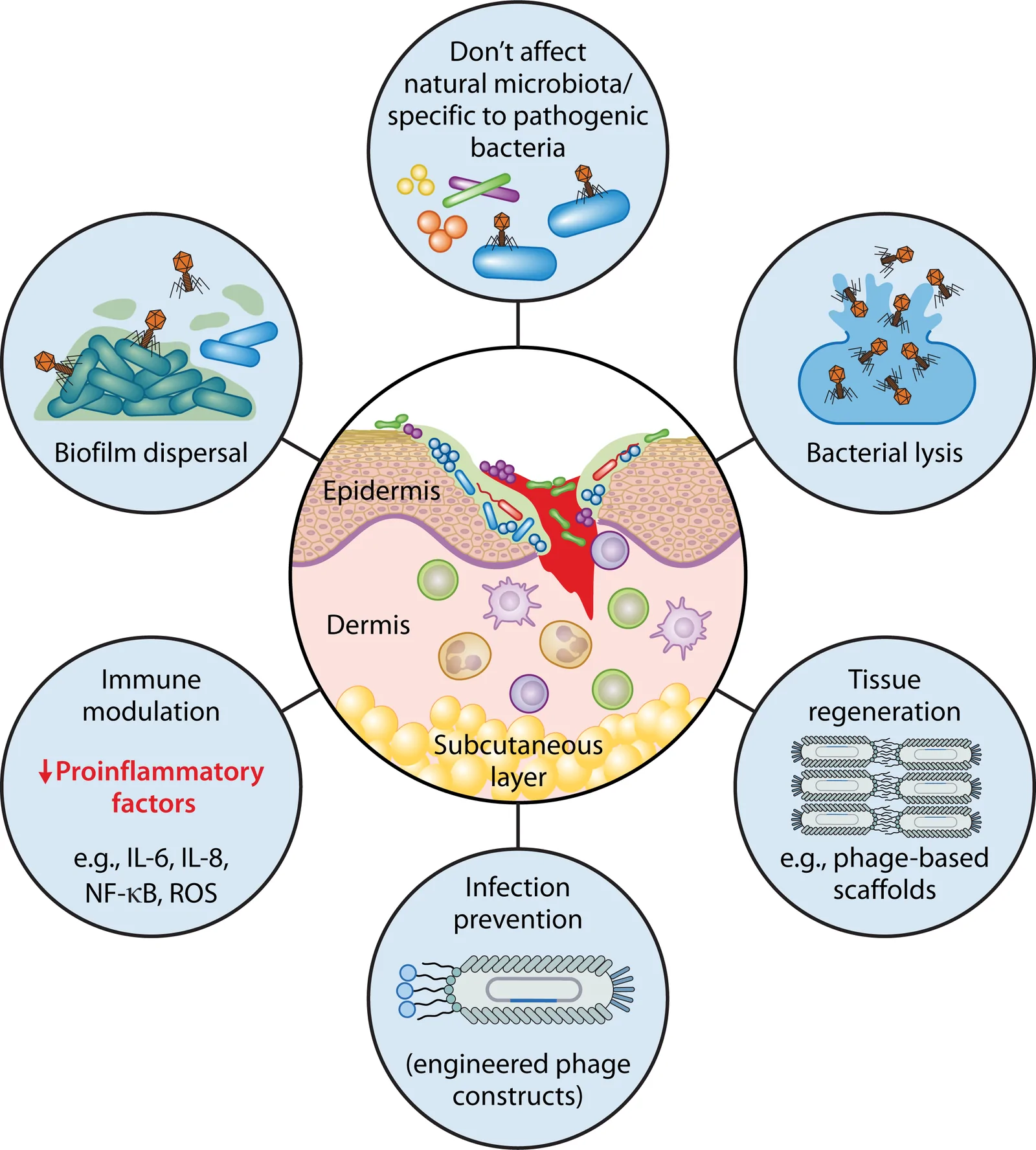
Multifactorial roles of natural and engineered bacteriophages in wound healing. Schematic representation of bacteriophage activities in infected skin wounds. Effects attributed to natural phages include selective bacterial targeting and lysis, biofilm dispersal, preservation of the commensal microbiome, and downregulation of pro-inflammatory mediators (e.g., IL-6, IL-8, NF-κB, ROS), collectively supporting immune modulation and restoration of a reparative wound microenvironment. Engineered phage-based approaches are represented by phage display-derived constructs for targeted infection prevention and phage-based scaffolds supporting tissue regeneration. Together, these mechanisms highlight the dual therapeutic potential of natural and engineered bacteriophages in infected wound management.

In the context of wound healing, it is also worth mentioning the emerging directions in phage applications. Beyond their antimicrobial and immunomodulatory roles, phages have also been explored as building blocks in regenerative medicine. Filamentous phages (e.g., M13, Pf1, Ff) possess nanoscale dimensions that enable self-assembly into nanowires and fibrous composites, potentially serving as scaffolds that mimic the native extracellular matrix (184). Phage-based scaffolds have been investigated for cartilage, bone, and neural tissue regeneration, and their biocompatibility and biodegradability make them attractive for future wound care applications (185–188). Such systems may support tissue repair by providing structural guidance while enabling localized and sustained delivery of therapeutic molecules. In addition, engineered phage surfaces can be functionalized with bioactive peptides, growth factors, or cell adhesion motifs to further enhance regenerative performance. In preclinical models, these functional modifications have been shown to promote cell migration, proliferation, and neovascularization at the wound site (188). The multifaceted roles of both natural and engineered phages in infected wounds, including biofilm disruption, selective bacterial lysis, modulation of inflammatory responses, infection prevention through phage-display-engineered constructs, and support of tissue repair through phage-based scaffolds, are schematically illustrated in Fig. 2.

## THE LINK BETWEEN PHAGE THERAPY AND MELANOMA TREATMENT

Cancer has long remained one of the most challenging diseases to treat, and its global burden continues to rise. Each year, it accounts for nearly 10 million deaths (189). Projections suggest that by 2040, the number of new cancer cases worldwide will increase by almost half, reaching 28.4 million (190). Despite remarkable progress in treatment, including chemotherapy, radiotherapy, targeted therapies, and immunotherapies, significant limitations persist, such as toxicity, high costs, relapse, and insufficient selectivity toward malignant cells (191). These challenges highlight the urgent need for novel approaches that are both more precise and less toxic.

Melanoma is a cancer type for which such novel approaches have particular significance. Although it accounts for only about 1% of all skin cancers, melanoma is the most lethal subtype, responsible for the majority of skin cancer-related deaths (192). Originating from melanocytes, the pigment-producing cells of the skin, melanoma is highly aggressive and metastasizes quickly, making delayed diagnosis particularly dangerous (193). While targeted therapies and immunotherapies have revolutionized melanoma treatment, resistance and relapse remain pressing challenges (194).

In this context, bacteriophages have emerged as an unexpected yet promising avenue of investigation. Beyond their established role in antibacterial therapy, phages have been shown to directly influence tumor cell behavior. Bacteriophage T4 and its mutant HAP1, selected for increased affinity toward melanoma cells, provide a particularly relevant model to explore such effects (195). *In vitro*, purified T4 and HAP1 bacteriophage preparations were shown to inhibit migration of both murine (B16) and human (Hs294T) melanoma cells on fibronectin, with no significant differences between the two phages. In addition, HAP1, but not T4, exhibited a statistically significant inhibitory effect on Hs294T migration on Matrigel, whereas neither phage significantly affected B16 migration on this matrix. These findings suggested that certain phage preparations can inhibit melanoma cell motility *in vitro*, with variations depending on cellular context and extracellular matrix components (196). These effects were independent of endotoxin contamination, as no response to LPS was observed, and residual LPS levels were minimal (196). The observed *in vitro* antimigratory activity may involve interference with RGD-binding integrins such as α(v)β(3) and α(5)β(1), which regulate adhesion, motility, and matrix remodeling in melanoma cells. This suggestion is based on the known involvement of these integrins in mediating cell-matrix interactions and their established roles in melanoma cell migration, as well as previous studies showing that disruption of RGD-integrin interactions can reduce motility and invasion (196, 197). Other adhesion molecules, including L1-CAM, VCAM-1, and CEACAM1, could also contribute, though direct interactions with phages remain unconfirmed (198).

While *in vitro* migration assays exclude the influence of the complex mammalian immune system, observations of antimetastatic effects *in vivo* suggested that host-mediated mechanisms may contribute to the enhanced efficacy of HAP1 (196). HAP1 was able to bind tumor cells more effectively than T4 and, in animal melanoma models, exhibited stronger antimetastatic activity, indicating a synergy between direct antimigratory effects and immune-mediated tumor cell clearance (196, 199). *In vivo*, components such as phagocytes and cytokine-mediated responses appear to play a critical role in enhancing tumor cell clearance and inhibiting metastasis, potentially through engagement of integrin-mediated adhesion pathways and modulation of immune signaling (197, 200). The combined effect of direct antimigratory activity and immune-mediated tumor cell elimination provides a plausible explanation for the superior antimetastatic efficacy of HAP1 observed in animal models compared to T4 alone. These findings indicate that phages can directly reduce melanoma cell motility without stimulating proliferation, highlighting a potential dual benefit for oncology patients: controlling infections while modestly limiting tumor cell migration. Further studies are needed to clarify the molecular mechanisms and evaluate effects across different phage types and normal immune cells (196).

Building on this intrinsic potential, researchers have further expanded phage applications in oncology through surface engineering and directed evolution technologies (191, 200). Phages are safe for eukaryotic cells and, thanks to their nanoscale size and modifiable surface, they can carry drugs, nucleic acids, or even tumor antigens directly to cancer cells. Their surfaces can be engineered to recognize specific tumor-associated proteins, offering a level of precision that traditional therapies often lack (201, 202). The key breakthrough that enabled this transition is phage display technology, introduced by George P. Smith in 1985 (203). This method allows the creation of vast peptide or antibody libraries displayed on the phage surface, followed by selection of those with the strongest affinity for a chosen target, such as a protein expressed on tumor cells (204, 205). This approach has enabled the identification of a wide range of targeting ligands and therapeutic constructs, from tumor-homing peptides to monoclonal antibodies, nanobodies, and CAR-T cells (206–209). As early as in the 1990s, Pasqualini and Ruoslahti (210) demonstrated that phage “biopanning,” a selection process in which phages displaying target-binding peptides are enriched, could yield peptides homing directly to tumors or their vasculature, interfering with angiogenesis (210). Later studies uncovered sequences with even greater selectivity for melanoma, for example, peptide C, which binds melanoma cells nearly 50 times more strongly than normal tissue and shows antitumor effects when linked to VEGFR-2 antagonists (211).

Beyond the identification of simple binding ligands, phage display has enabled the development of increasingly sophisticated therapeutic constructs. One striking example is the nanobody C5, designed to recognize GRP78, a stress-induced chaperone protein expressed on melanoma cell surfaces. When fused with a truncated *Pseudomonas* exotoxin (PE38), this recombinant immunotoxin (C5-PE38) not only killed melanoma cells but also activated antitumor immunity. Combined with PD-1 checkpoint inhibition, it significantly reduced tumor growth and metastasis (212). Similarly, phage-display-derived TCR-like antibodies have been engineered into CAR-T cells that recognize melanoma-associated antigens presented by MHC molecules, achieving targeted tumor killing with minimal off-target toxicity (213). Other applications include bispecific antibodies and engineered antibody constructs targeting melanoma proteins such as GPNMB or VCAM-1, some of which have been identified or optimized using phage display technologies (214), as well as humanized monoclonal antibodies against growth factors like FGF2 that block melanoma cell proliferation and migration (215).

Importantly, phage display is also transforming melanoma diagnostics and enabling theragnostic strategies based on the molecular recognition of tumor-associated markers. Peptides such as CY12-RP2, selected against podoplanin (PDPN), a protein overexpressed in melanoma and associated with tumor invasiveness, demonstrate how precise targeting of diagnostic markers can simultaneously yield therapeutic benefits. By binding PDPN, CY12-RP2 not only inhibited tumor progression but also enhanced immune responses (216). Meanwhile, PD-L1-binding peptides, like CLP002, have been developed into radiolabeled PET tracers, enabling precise imaging of melanoma and other cancers in preclinical models (217). Such approaches illustrate how ligands identified through phage display can serve both as molecular imaging tools and as functional modulators of tumor biology.

The use of phages in oncology reflects an interesting progression: from viruses first recognized for infecting bacteria to potentially useful therapeutic and diagnostic tools. In melanoma, where treatment challenges remain significant, phage display has enabled the development of targeted therapeutic and diagnostic strategies. Rather than being viewed solely as biological curiosities, phages are increasingly investigated as platforms for cancer research. Their selectivity, capacity for engineering, and generally favorable safety profile make them a valuable area of study for precision medicine, and ongoing research continues to explore their clinical potential.

## RESEARCH ISSUES AND PERSPECTIVES

The data and analyses presented in this review clearly indicate that phage therapy is a promising and biologically sound therapeutic strategy for the treatment of skin and soft tissue infections, particularly in the context of increasing antibiotic resistance, the ability of pathogens to form biofilms, and wound-healing challenges. The high specificity of phages, the ability to eliminate multidrug-resistant pathogens, and their minimal impact on the skin microbiome distinguish phage therapy from the treatment of choice, the use of broad-spectrum antibiotics, making it an attractive alternative or complementary treatment option. At the same time, it is important to emphasize that despite numerous promising results from *in vitro* and animal model studies, available data from clinical trials are still limited, which hinders a clear assessment of phage therapy’s effectiveness in clinical practice, though strongly emphasizing its safety.

Across all pathogens reviewed, a recurring limitation is the scarcity of robust clinical data. Most available evidence derives from *in vitro* or animal studies, with clinical experience confined largely to case reports, small cohort studies, and compassionate-use programs. An overview of registered clinical trials specifically addressing SSTIs is provided in Table 3. Heterogeneity in study designs, phage preparations, dosing regimens, and delivery systems further complicates cross-study comparisons. Consequently, well-designed, adequately powered randomized controlled trials are needed for all major skin pathogens discussed in this review. Unifying criteria for assessing the effectiveness of phage therapy, encompassing both microbiological parameters and clinical indicators of wound healing, will be particularly important to allow comparison of results across studies and facilitate their interpretation by regulatory agencies.

One of the key factors determining the success of phage therapy in dermatology is the method of phage delivery to the site of infection. Undoubtedly, the clinical effectiveness of bacteriophage-based preparations depends not only on the selection of specific phages but also on their stability and, inevitably, on the use of an appropriate number of virions, as well as on formulations that ensure their activity and dynamic access to the wound environment. In this context, advanced platforms composed of biomaterials, such as hydrogels, adhesive dressings, nanoparticle-based systems, or electrospun scaffolds, seem particularly promising. These platforms enable controlled phage release, increase their retention at the site of application, and protect against inactivation.

In the future, clinical applications of phage therapy for skin infections will likely evolve toward a precise, personalized, and combined approach. Nevertheless, one should remember that to ensure rapid medical intervention and to provide phage-based therapeutics to patients, centers undertaking such therapy must have large collections of well-characterized bacteriophages, as well as developed procedures for phage multiplication, purification, and formulation. Otherwise, patients could wait for the therapy dangerously long, their health could deteriorate, and consequently, the effectiveness of the therapy would be reduced.

Undoubtedly, the use of phage cocktails with a broader spectrum of activity than single phages and the combination of phages with antibiotics can increase treatment efficacy, particularly in eliminating biofilm structures and skin infections caused by multiple bacterial species. Increasing evidence also indicates that the benefits of phage therapy extend beyond pathogen elimination and may include modulation of the inflammatory response and improvement of the wound microenvironment, which promotes healing and tissue regeneration. At the same time, it is important to emphasize that future research must systematically address existing limitations of phage therapy, including the risk of bacterial resistance to phages, neutralization by the host immune system, resistance to changing wound conditions, and the complex nature of chronic infections. Rigorous characterization of phage genomes intended for clinical use and the standardization of their production, purification, and quality control processes in accordance with GMP requirements are also essential. Such an approach should determine clinical success, but unfortunately, it carries limitations due to the increased cost of the therapy, which may significantly limit patient access.

Unifying criteria for assessing the effectiveness of phage therapy, including both microbiological parameters and clinical indicators of wound healing, is particularly important. This will allow for comparison of results across studies and facilitate their interpretation by regulatory agencies. Broadly speaking, bacteriophages are increasingly being viewed not only as tools for combating bacterial infections, but also as multifunctional technological platforms. The use of phages in modulating the skin microbiome, treating acne, engineering biomaterials, and utilizing phage display technology for the diagnosis and treatment of skin cancers, including melanoma, indicates the dynamic development of this field. Many of these new research directions highlight the potential of phages as a component of future precision medicine.

In summary, phage therapy in dermatology has a sound biological basis and a growing number of positive experimental results, particularly in the treatment of chronic wounds and antibiotic-resistant infections. To introduce phage therapy into standard clinical procedures, it is imperative to ensure, above all, progress in clinical trial design, standardized production, and the development of effective and stable phage delivery systems that will translate their biological potential into reproducible and measurable therapeutic benefits.
